# Association of the *IL-10* Gene Family Locus on Chromosome 1 with Juvenile Idiopathic Arthritis (JIA)

**DOI:** 10.1371/journal.pone.0047673

**Published:** 2012-10-18

**Authors:** Ebun Omoyinmi, Paola Forabosco, Raja Hamaoui, Annette Bryant, Anne Hinks, Simona Ursu, Childhood Arthritis Prospective Study (CAPS), BSPAR study group, Childhood Arthritis Response to Medication Study (CHARMS), Lucy R. Wedderburn, Wendy Thomson, Cathryn M. Lewis, Patricia Woo

**Affiliations:** 1 Department of Immunology and Molecular Pathology, University College London, London, United Kingdom; 2 Istututo di Genetica delle Popolazioni, CNR, Sassari, Italy; 3 Arthritis Research UK Epidemiology Unit, Manchester Academic Health Science Centre, The University of Manchester, Manchester, United Kingdom; 4 Rheumatology Unit, Institute of Child Health, University College London, London, United Kingdom; 5 King’s College London, Department of Medical and Molecular Genetics, London, United Kingdom; 6 King’s College London, MRC SGDP Centre, Institute of Psychiatry, London, United Kingdom; University of Freiburg, Germany

## Abstract

**Background:**

The cytokine *IL-10* and its family members have been implicated in autoimmune diseases and we have previously reported that genetic variants in *IL-10* were associated with a rare group of diseases called juvenile idiopathic arthritis (JIA). The aim of this study was to fine map genetic variants within the *IL-10* cytokine family cluster on chromosome 1 using linkage disequilibrium (LD)-tagging single nucleotide polymorphisms (tSNPs) approach with imputation and conditional analysis to test for disease associations.

**Methodology/Principal Findings:**

Fifty-three tSNPs were tested for association between Caucasian paediatric cohorts [219 systemic JIA (sJIA), 187 persistent oligoarticular JIA (pOJIA), and 139 extended OJIA (eOJIA) patients], and controls (Wellcome Trust control cohort, WTCCC2). Significant association with sJIA was detected at rs1400986 in the promoter of *IL-20* (odds ratio 1.53; 95% CI 1.21–1.93; p = 0.0004), but in no other subtypes. Imputation analysis identified additional associated SNPs for pOJIA at *IL-20* and *IL-24*, including a rare, functional, missense variant at *IL-24* with a p = 0.0002. Penalised logistic regression analysis with HyperLasso and conditional analysis identified several further associations with JIA subtypes. In particular, haplotype analysis refined the sJIA association, with a joint effect at rs1400986 and rs4129024 in intron 1 of *MAPKAPK2* (p = 3.2E−5). For pOJIA, a 3-SNP haplotype including rs1878672 in intron 3 of *IL-10* showed evidence for association (p = 0.0018). In eOJIA, rs10863962 (3′UTR of *FCAMR*) and rs12409577 (intron of IL-19) haplotype showed some evidence of association (p = 0.0003).

**Conclusions:**

This study supports previous association of *IL-20* with sJIA. Haplotype analyses provided stronger association signals than single point analyses, while a penalised logistic regression approach also suggested multiple independent association signals. Replication studies are required to confirm or refute these findings. The results indicate that combined effects with unknown/rare variants remain to be characterised in JIA, and represent a possible example of synthetic association in this region.

## Introduction

Juvenile idiopathic arthritis (JIA) represents a heterogeneous group of childhood arthritides that persist for more than 6 weeks with an onset before the age of 16 years [1]. JIA affects approximately 1∶1000 children. According to the International League of Associations for Rheumatology (ILAR) classification system, the disease is divided into 7 distinct clinical subtypes [1]. The groups investigated in this study are systemic JIA (sJIA) comprising approximately 10% of all JIA, and oligoarthritis (OJIA) which comprise approximately 40% of all JIA. OJIA is further subdivided into persistent and extended oligoarticular JIA (referred to in this report as pOJIA and eOJIA respectively). If a child has 4 or fewer joints involved at the time of study and has this for at least 6 months they are considered to have pOJIA. However if they “extend” and have more than 4 joints involved after first 6 months of disease, they are considered to have eOJIA (approximately 50% of OJIA). The systemic features of sJIA make this subtype clinically distinct from the other subtypes of JIA [2]. Evidence for differences between these subtypes is strengthened by a recent study using mRNA profiling which showed distinct patterns of gene expression in sJIA, compared with oligoarticular JIA [3]. The characteristic immunological profile of sJIA is the activation of innate immunity genes, whereas the characteristic feature of OJIA is the strong association with the HLA class I & II loci, which are supported by family studies [4].

Interleukin-10 (*IL-10*) is an important immunoregulatory cytokine with anti-inflammatory properties. It is produced by several cells including activated monocytes/macrophages, and subsets of regulatory B and T cells. *IL-10* is known to suppress the release and function of a number of proinflammatory cytokines, including *IL-1β*, *TNF-α*, and *IL-6* [5]. The gene family of *IL-10* consisting of *IL-19, IL-20*, and *IL-24* are located within a highly conserved cytokine gene cluster on chromosome 1q32. Like the *IL-4* cytokine gene cluster, recent evidence from the mouse *IL-10* gene family cluster suggest that there is coordinate regulation of these cytokines by distal regulatory elements spanning the locus [6]. Associations with cytokine genes, in particular *IL-10*, are also different between subtypes of JIA. In our previous study of the proximal *IL-10* haplotypes formed by 3 single-nucleotide polymorphisms (SNPs) at -1082A/G (rs1800896), -819C/T (rs3021097), and -592A/C (rs1800872), we found an increased frequency of the ATA haplotype in patients with eOJIA [7]. It was demonstrated in the same study that the ATA haplotype found in healthy individuals is associated with low *IL-10* production in LPS stimulated whole blood culture and a weaker transcriptional activity than GCC haplotype. *IL-10* production is also lower in parents of children with eOJIA compared with those of children with pOJIA, and have increased frequency of the ATA haplotype when compared to controls [8].

Four SNPs (two in the *IL-10* gene; one in the *IL-19* gene; and one in the *IL-20*) from three members of the *IL-10* gene family were examined in sJIA patients in one of our previous candidate gene association studies [9]. Significant differences in allele frequency were observed between cases and controls, for both *IL-10*-1082 (rs1800896) (p = 0.031) and *IL-20*-468 (rs1400986) (p = 0.028). Furthermore, examination of the two-SNP haplotypes revealed stronger evidence for association (global p = 0.0006).

These genetic findings from our earlier studies, together with the known role of *IL-10* in controlling inflammation, led us to consider the extended family of *IL-10* related cytokines as candidate genes involved with sJIA as well as OJIA. In this study we investigated disease association of the *IL-10* family of cytokines and flanking genes in linkage disequilibrium (identified using the Tagger function in Haploview), in a cohort comprising three different JIA subtypes. The OJIA samples represent a different UK cohort to our previous study. For sJIA, 122 of the 219 samples were previously genotyped therefore this present study is an extension of the previous cohort. Using tagging SNPs (tSNPs) that cover a 390 Kb region, we used three different strategies to explore the genetic contribution of the *IL-10* region to JIA. Firstly, we tested genotyped tSNPs for association with JIA subtypes using WTCCC2 controls in a single-point analysis. Secondly, we imputed SNPs in the 1000 genomes project and tested for association to determine whether ungenotyped SNPs, particularly rarer variants, showed stronger association for JIA. In order to identify independent association signals within the region, we also applied a penalized logistic regression approach for all typed or imputed SNPs. Thirdly, in an exploratory analysis to determine how multiple SNPs are associated with JIA subtypes, we perform conditional analyses to fine map the region for haplotype-based signals of association. These methods enable us to explore the hypothesis of synthetic association in JIA in this region. To further explore genetic effects in this region, expression studies were undertaken using HapMap samples.

## Results

Genotype data from a total of 545 JIA patients (219 sJIA, 187 pOJIA, 139 eOJIA) passed quality control with a call rate of >90%. There was 100% concordance in genotype calling of duplicate samples. Among the 68 tSNPs selected, 5 failed genotyping and data for 53 tSNPs were available from WTCCC2 healthy individuals genotyped either by Affymetrix, or Illumina, or both. Genotype data were available for 5723 WTCCC2 subjects, who were used in the single point analysis. A subset of 4813 WTCCC2 controls had complete genotyping for the 53 tSNPs, and were therefore used in the imputation, conditional and haplotype analyses. All tSNPs included in the analysis had genotype distributions in Hardy-Weinberg equilibrium (p-values >0.01).

Different levels of significance were applied as appropriate for each analysis strategy. For the single-point analysis of 53 SNPs, Nyholt’s method [10] calculated the SNPs were equivalent to 30.9 independent tests within the region based on the LD pattern in the WTCCC2 controls, giving a Bonferroni corrected p-value of 0.00166. The imputed SNPs were equivalent to 320 independent tests, and a corresponding p-value of 0.00016, to retain the type 1 error at 0.05. For the exploratory conditional analysis, no equivalent methods to determine an appropriate multiple testing correction exist. We therefore used a threshold of *suggestive* significance of p = 0.0189 = 1/53 to determine which SNPs should be added to the conditional analysis (this p-value is the level expected to arise once in the analysis of 53 SNPs in this region).

### Systemic JIA [sJIA]

**Figure 1:**
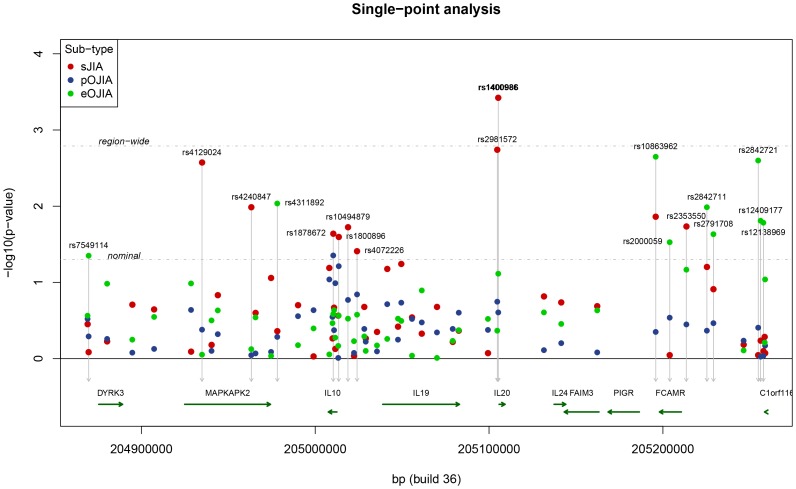
Single point results for sJIA, pOJIA, and eOJIA. Point colours indicate the different JIA subtypes. Thresholds are indicated for nominal (p = 0.05) and region-wide significance (p = 0.00166).

**Figure 2:**
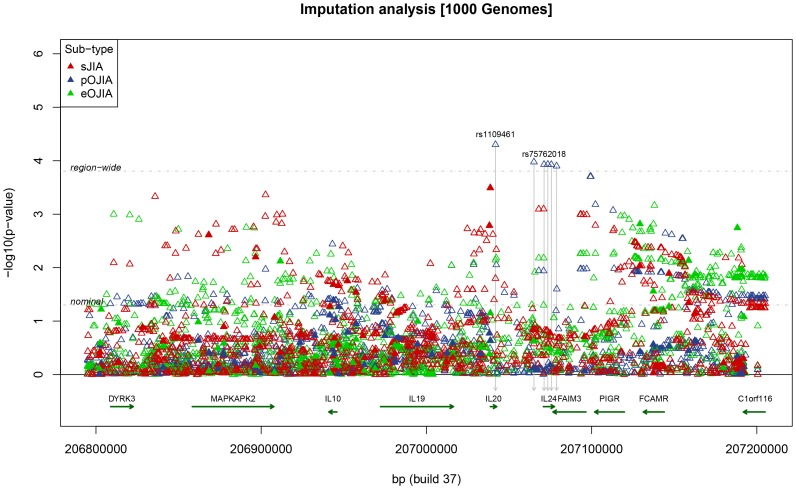
Imputation analysis for sJIA, pOJIA, and eOJIA, showing nominal (p = 0.05) and region-wide significance at p = 00016. For imputed SNPs a cut-off of Rsq >0.3 was used (see text).

This study of 219 sJIA cases detected significant association at rs1400986, a promoter variant of *IL-20* at position −468 (Figure 1) (p = 0.0004; OR = 1.53, 95% CI 1.21–1.93; Table 1). Association at this SNP was previously described by Fife et al [9] in a smaller, but overlapping cohort of sJIA cases. This tSNP gave the strongest single-marker association signal across all SNPs and all JIA subtypes. In the imputation analysis, no SNP exceeded the region-wide p-value for significance (Figure 2; Supplementary Table S1). Penalised logistic regression retained 4 SNPs (all imputed) in the resulting mode (Supplementary Figure S2), although the same evidence for association (log-posterior) was observed for several different groups of retained SNPs (data not shown), indicating that several different choices of SNPs could be used interchangeably.

**Table 1:** Results of single point analysis for JIA subtypes and 5723 WTCCC2 controls, showing tSNPs associated at p<0.05. MAF = minor allele frequency.

			WTCCC	sJIA (N = 219)				pOJIA (N = 187)				eOJIA (N = 139)			
SNP	gene	allele	MAF	freq*	p	OR	95% CI	freq*	p	OR	95% CI	freq*	p	OR	95% CI
rs7549114	*DYRK3*	A,G	0.15	0.15	0.8201	1.03	[0.79;1.35]	0.14	0.5107	0.90	[0.67;1.22]	0.10	0.0445	0.67	[0.46;0.99]
rs4129024†	*MAPKAPK2*	A,G	0.23	0.17	0.0027	0.68	[0.53;0.88]	0.21	0.4173	0.90	[0.7;1.16]	0.23	0.8837	0.98	[0.74;1.3]
rs4240847	*MAPKAPK2*	C,A	0.27	0.21	0.0103	0.74	[0.58;0.93]	0.26	0.9016	0.99	[0.78;1.25]	0.26	0.7493	0.96	[0.73;1.26]
rs4311892†	*MAPKAPK2*	G,A	0.26	0.27	0.4342	1.09	[0.88;1.35]	0.27	0.5169	1.08	[0.86;1.36]	0.32	0.0092	1.40	[1.09;1.82]
rs1878672†	*IL10*	G,C	0.48	0.53	0.0229	1.25	[1.03;1.51]	0.53	0.0443	1.24	[1.01;1.52]	0.51	0.2600	1.15	[0.9;1.46]
rs1800896†	*IL10*	A,G	0.48	0.53	0.0253	1.25	[1.03;1.51]	0.53	0.0610	1.22	[0.99;1.5]	0.51	0.2723	1.14	[0.9;1.45]
rs10494879	*IL10*	G,C	0.45	0.39	0.0188	0.79	[0.65;0.96]	0.41	0.1690	0.86	[0.7;1.07]	0.41	0.2987	0.88	[0.69;1.12]
rs4072226	*IL10*	T,C	0.45	0.40	0.0389	0.81	[0.67;0.99]	0.41	0.1439	0.86	[0.69;1.06]	0.41	0.2653	0.87	[0.68;1.11]
rs2981572∧	*IL20*	G,T	0.37	0.44	0.0018	1.36	[1.12;1.65]	0.40	0.1785	1.16	[0.94;1.43]	0.39	0.4301	1.10	[0.86;1.41]
rs1400986†	*IL20*	T,C	0.15	0.21	**0.0004**	1.53	[1.21;1.93]	0.17	0.2477	1.17	[0.89;1.54]	0.19	0.0766	1.32	[0.97;1.78]
rs10863962†	*FCAMR*	T,C	0.26	0.32	0.0137	1.30	[1.05;1.59]	0.28	0.4465	1.09	[0.87;1.38]	0.35	0.0022	1.48	[1.15;1.9]
rs2000059	*FCAMR*	A,G	0.11	0.11	0.8961	1.02	[0.75;1.39]	0.09	0.2895	0.82	[0.57;1.18]	0.07	0.0295	0.59	[0.36;0.95]
rs2353550†	*FCAMR*	G,A	0.43	0.48	0.0185	1.26	[1.04;1.52]	0.45	0.3547	1.10	[0.9;1.36]	0.48	0.0680	1.25	[0.98;1.58]
rs2842711†	*FCAMR*	T,A	0.35	0.39	0.0627	1.21	[0.99;1.47]	0.37	0.4308	1.09	[0.88;1.35]	0.42	0.0103	1.37	[1.08;1.74]
rs2791708	*FCAMR*	C,T	0.36	0.39	0.1229	1.17	[0.96;1.42]	0.38	0.3414	1.11	[0.9;1.37]	0.42	0.0233	1.32	[1.04;1.68]
rs2842721†	*C1orf116*	A,G	0.23	0.23	0.9007	1.02	[0.81;1.27]	0.21	0.3928	0.90	[0.7;1.15]	0.31	0.0025	1.49	[1.15;1.93]
rs12409177	*C1orf116*	T,C	0.15	0.16	0.5817	1.08	[0.83;1.4]	0.14	0.9408	0.99	[0.74;1.33]	0.20	0.0155	1.45	[1.07;1.95]
rs12138969†	*C1orf116*	A,G	0.15	0.15	0.7951	1.04	[0.79;1.35]	0.14	0.9214	0.99	[0.74;1.32]	0.20	0.0165	1.44	[1.07;1.94]

Significant evidence of association was detected at rs1400986 for sJIA.

* Allele frequencies of the first allele (minor allele) is indicated.

∧ rs2981572 is from PGA.

† tSNPs examined for correlation between lymphoblastoid mRNA expression and HapMap genotypes.

**Figure 3:**
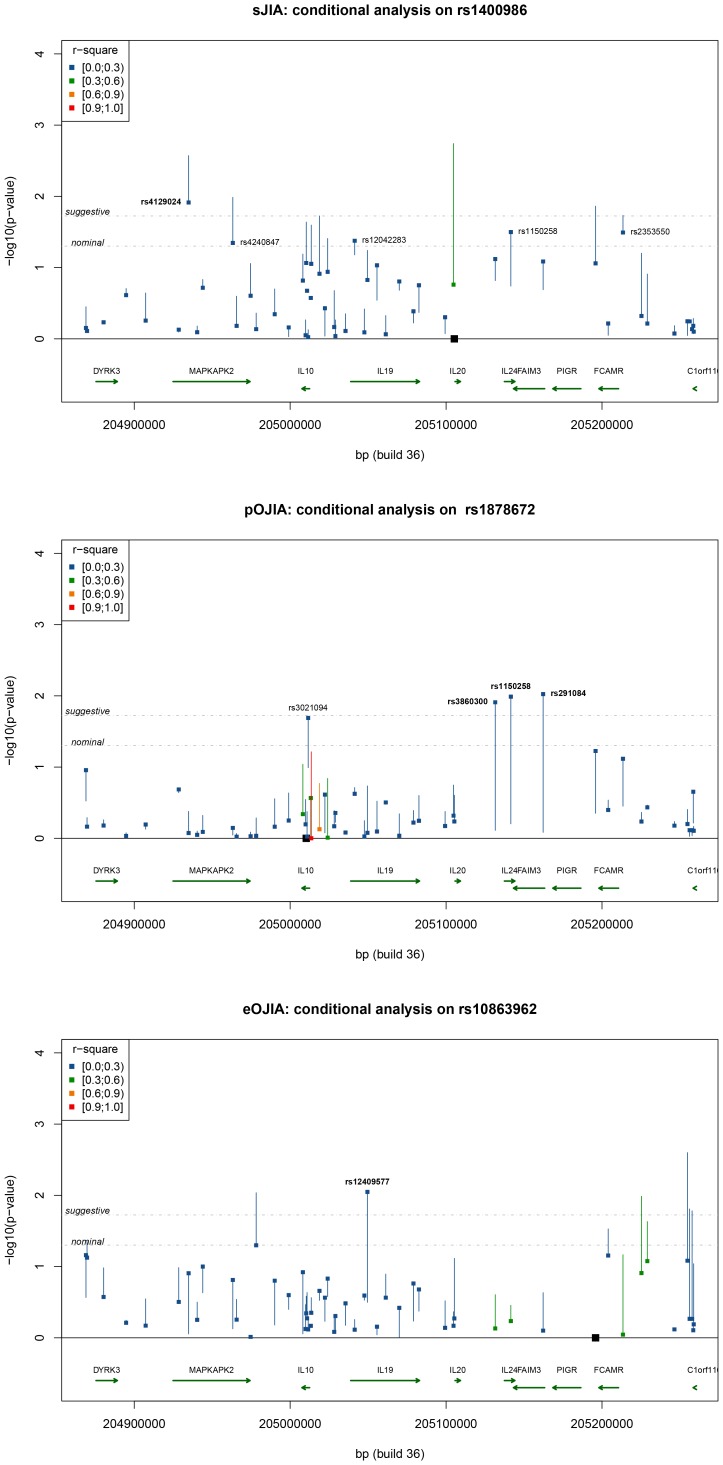
Conditional analysis on the most significant SNPs for each JIA subtype. For each SNP, the square represents the p-value after adjusting for the effect of the conditioned SNP, while the other end of the line shows the p-value of a single-locus analysis, prior to conditioning. Colours show the range of r^2^ between the conditioned SNP and the tested SNP as indicated in the insert. Location of the conditioned SNPs is indicated by a black square. Thresholds are indicated for nominal (p = 0.05), suggestive (p = 0.0189) thresholds, with tSNPs above suggestive threshold indicated in bold.

**Figure 4:**
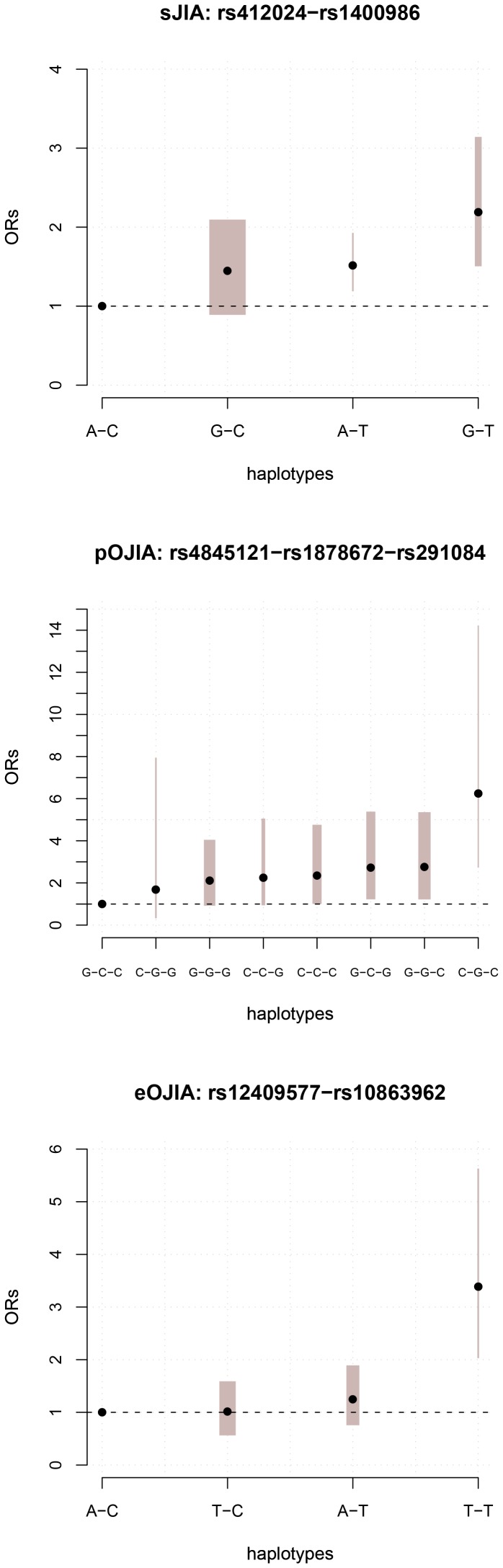
Odds ratios for each haplotype in the best-fitting model for each JIA subtypes, with 95% CI (grey band, with width proportional to haplotype frequency in controls).

In further analysis of the region, we conditioned on rs1400986 and analysed each SNP to detect further signals of association. Decreased evidence for association was observed at many SNPs, but one SNP, rs4129024, remained associated at a suggestive level of significance (Figure 3). After conditioning on both rs1400986 (*IL-20*) and rs4129024 (*MAPKAPK2*), no additional tSNP showed suggestive evidence for association. We then examined in more detail the combination of the risk alleles of rs1400986 and rs4129024 tSNPs. The SNP-based model provided the best fitting model (p = 3.2E−5; Table 2), indicating independent contributions to risk from these two SNPs, with no interactions between them. The haplotype of risk alleles [G-T] at rs4129024 and rs1400986 confers an OR of 2.19 for sJIA (95% CI: 1.55–3.10; p = 5.6E−06) compared to the lowest-risk alleles [A-C] (Figure 4); G-T had a frequency of 19% in sJIA cases compared to 12% in WTCCC2 controls.

**Table 2:** Conditional analysis, conditioning on the SNP with the strongest evidence of association, and adding additional SNPs into the model.

JIA sub-	Conditioning SNPs	Most significant SNP(s)	SNP p-value in	Final model p-values	
phenotype			conditioning model	Haplotypemodel	SNP model (joint effect)
**sJIA**	–	rs1400986	0.0004	–	–
	rs1400986	rs4129024	0.0122	–	–
	rs1400986–rs4129024	–	–	0.0001 (0.00014)	3.2e−05 (4e−05)
**pOJIA**	–	rs1878672	0.0443	–	–
	rs1878672	rs3860300	0.0123	–	–
		rs1150258	0.0103	–	–
		rs291084	0.0095	–	–
	rs1878672–rs291084	rs4845121	0.0068	–	–
	rs1878672–rs291084–rs4845121	–	–	0.0018 (0.00184)	0.0453 (0.04077)
**eOJIA**	–	rs10863962	0.0022	–	–
	rs10863962	rs12409577	0.0090	–	–
	rs10863962–rs12409577	–	–	0.0003 (0.00025)	0.0014 (0.00156)

For each JIA subtype, the most significant SNP from single point analysis (Table 1) was used to start the conditional analysis, and then the most significant SNP achieving at least suggestive significance (p<0.0189) was added into the conditional model until no further SNPs were identified. In parentheses are reported the empirical p-values obtained by running 100,000 permutations.

### Persistent Oligoarticular JIA [pOJIA]

No SNP showed significant evidence for association for pOJIA in the analysis of genotyped tSNPs (Table 1). In the imputation analysis, one SNP in *IL20* and five SNPs in (or near) *IL-24* exceeded the p-value threshold adjusted for multiple testing of imputed SNPs. However these SNPs are all rare (with imputed frequency <0.001). The Rsq quality metric for imputation in MACH was ∼0.4 which exceeded our chosen threshold of 0.3 but reflects the difficulty in imputing rare variants that are only weakly tagged by the genotyped SNPs. Full results of the imputation analysis in this region are included in Supplementary Table S1. Penalised logistic regression identified 14 SNPs (all imputed) in the mode with highest log-posterior. The retained SNPs were those in the IL-4 region showing significance above region-wide thresholds, and additional SNPs located in the *FAIM3-PIGR-FCAMR* region with lower association p-values (Supplementary Figure S2).

Exploratory haplotype analysis was performed, initially conditioning on tSNP rs1878672 in intron 3 of *IL-10*, which showed the strongest association with pOJIA (p = 0.0443). Conditioning on this tSNP, three further tSNPs (rs3860300, rs1150258, and rs291084) reached suggestive evidence for association (p<0.0189; Figure 3). These tSNPs are all located in the *IL-24/FAIM3* region and in strong LD with each other, but not with rs1878672 (all pairwise r^2^>.85, see Supplementary Figure S1). The conditional analysis on rs1878672 substantially increased the evidence for association at these tSNPs, since none achieved even nominal association when analysed singly (Table 1). When conditioning on rs1878672 and rs291084, there was additional evidence for association below suggestive p-value threshold at rs4845121, which is located 139 Kb downstream of *IL-10* in the promoter region of the *DYRK3*. Conditional analysis of these three tSNPs identified no further associations. Haplotype analysis of these SNPs (rs4845121–rs1878672–rs291084) showed several haplotypes with elevated risk. The highest risk was for a C-G-C haplotype conferring an OR of 6.25 (95% CI: 2.76–14.19) compared to the lowest-risk haplotype [G-C-C] under the full haplotype model (p = 0.0002; Figure 4). The frequency of the C-G-C haplotype was higher in cases (6%) than controls (2%).

### Extended Oligoarticular JIA [eOJIA]

No SNP showed association meeting the Bonferroni threshold for significance in eOJIA, with the strongest evidence for association occurring at tSNP rs10863962 in *FCAMR* (p = 0.0022; Table 1). As for sJIA, the imputation analysis did not for any SNP reached significance at the region-wide p-value (Figure 2; Supplementary Table S1). Penalised logistic regression retained 14 SNPs (all imputed) in the resulting mode (Supplementary Figure S2), but the same log-posterior of the mode was observed for several combinations of retained SNPs.

Conditional analysis on the most significant tSNP (rs10863962) reduced the evidence for association at SNPs in *MAPKAPK2* and around *FCAMR/C1orf116*, but there was an increase in significance level to above suggestive threshold for one tSNP, rs12409577, located in the intron of *IL-19* (Figure 3). A conditional analysis on rs10863962 and rs12409577 detected no additional evidence for association. The full haplotype model in UNPHASED gave for these two tSNPs (p = 0.0003) fitted significantly better than the SNP model (Table 2). Under this model the combination of the risk alleles [T-T] resulted in an OR = 3.40, (95% CI 2.01–5.61; p = 7.35E−5 compared to the lowest-risk haplotype [A-C] (Figure 4). The frequency of the [T-T] haplotype was higher in cases (9%) than controls (3%).Our previous disease association study revealed a significant increase in the frequency of the homozygote genotype (ATA/ATA) of *IL-10* haplotype A-T-A (rs1800896–rs1800871–rs1800872) in 78 patients with eOJIA compared to 86 pOJIA [7]. In this current study, only rs1800896 and rs1800871 were typed, since rs1800872 is in complete LD with rs1800871. We did not observe a significant difference in the frequency of single alleles, the 2-marker A-T (rs1800896–rs1800871) or genotype distribution between patients with oligoarticular JIA when compared with controls. However, the frequency of the homozygote AT/AT genotype was higher in eOJIA (7.2%) than pOJIA (3.7%) as previously reported. The numbers of patient possessing the AT/AT homozygous genotypes were too small for statistical analysis.

### Association of Genotype with Gene Expression

We used the publicly available lymphoblastoid expression data with HapMap genotypes [11] to obtain preliminary functional information on the effect of tSNPs on mRNA expression, for 10 tSNPs with high risk of disease alleles (OR >1) from the single SNP analysis (Table 1). The result of linear regression analysis between the tSNP and HapMap expression data are shown in Table 3. The tSNPrs4129024 showed a significant correlation between genotype and expression of a *MAPKAPK2* isoform (NM_004759; p = 0.0027). The G allele has decreased expression of this *MAPKAPK2* isoform, and shows some evidence of increased risk of sJIA (p = 0.0027; Table 1). No other SNPs tested for expression reached the Bonferroni corrected p-value threshold, but several SNPs showed nominal evidence of significance (p<0.05; Table 3).

**Table 3:** Linear regression analysis between tSNPs and HapMap lymphoblastoid cell line expression levels *of MAPKAPK2, IL-10, IL-20, FCAMR* and *C1orf116*.

Gene	Significant tSNP	Location	Disease Associated allele*	Effect on Expression level		Linear regression p-value‡
***MAPKAPK2*** ** (NM_004759)** †	rs4129024	Intron 1	G	Decreased	**0.003**	
	rs4311892	3.94 kbdownstream	G	No effect	0.193	
***MAPKAPK2*** ** (NM_032960)** †	rs4129024	Intron 1	G	No effect	0.066	
	rs4311892	3.94 kbdownstream	G	No effect	0.055	
***IL-10***	rs1878672	Intron 3	G	Decreased	**0.022**	
	rs1800896	−1082 bp	A	Decreased	**0.011**	
***IL-20***	rs1400986	−468 bp	T	No effect	0.404	
***FCAMR***	rs10863962	2.24 kbdownstream	T	Increased	**0.009**	
	rs2353550	−3.34 kbupstream	G	Increased	**0.005**	
	rs2842711	−15.19 kbupstream	T	No effect	0.655	
***C1orf116***	rs2842721	3.66 kbdownstream	A	No effect	0.187	
	rs12138969	705 bpdownstream	A	No effect	0.609	

* Allele with increased frequency in children with JIA compared with controls. This corresponds to the minor alleles for all except rs4129024 where the frequency of major allele G is higher in cases than controls.

† Two transcript encoding two different isoforms have been found for this gene. They code for the same number of exons but differ at the 3′UTR region.

‡ The linear regression model was used to evaluate the association between log2-transformed expression values and genotypes of tSNPs. Associations reaching nominal significance (p<0.05) are shown in bold. One SNP reached the Bonferroni corrected threshold for 12 tests of 0.0042 = 0.05/12.

## Discussion

This study has performed an in depth candidate region analysis of the IL10 gene family in JIA subtypes. In the era of genome wide association studies (GWAS), there is still a place for the comprehensive mapping exercise reported here, as the density of genotyped SNPs exceeds that available in most genome wide chips, including ones employed in GWAS of JIA [12]. Fine mapping of the *IL-10* gene family has led to the identification of novel genes associated with subtypes of JIA. Since it is known that the tagging method performs poorly in predicting rare variants [13], we additionally explored the genomic region through imputation and through haplotype based and also penalized logistic regression based analyses. These analyses enable us to explore the hypothesis of “synthetic association” [14], [15], where signals of common non-functional SNPs outside of coding regions of a candidate gene may be the result of a combination of rare coding/functional variants with stronger effects present on the haplotype. Other studies have obtained stronger association signals upon combining tSNPs identified after conditioning on the primary signal, as carried out in this study, suggesting underlying, unidentified variant(s) that contribute to susceptibility in the region [16].

In this JIA study, we have evidence from conditional analysis in sJIA that there are likely to be multiple variants contributing to susceptibility: one in *IL-20* and another in *MAPKAPK2*. For persistent oligoarticular JIA, we observed that nominal evidence for association with rs1878672 (intron 3 of *IL-10*) is increased when considered in a 3-marker haplotype analysis. In the case of eOJIA, variants in 2 regions appear to contribute to susceptibility (*IL-19* and *FCAMR/C1orf116*), with some evidence of a haplotype effect across SNPs. Results from the HyperLasso method, that implements a penalised logistic regression analysis, also showed that subsets of SNPs were associated with the different JIA subtypes. Comparing results across these analyses is difficult since the methods are so different, but both analyses indicate that there is a complex pattern of genetic susceptibility to JIA in this region.

A major limitation of our study is that only modest sample sizes of cases can be obtained for this rare, heterogeneous disease, and we are further hindered by the lack of replication cohort. The study design focused on obtaining the largest possible sample size to detect association in a hypothesis-generating study, and further studies will need to be performed to refute or to confirm the associations detected here. An additional limitation of this study is that the identification of tSNPs and SNPs tagged was done predominantly through the use of HapMap and thus the experimental design relies on the completeness of the data. 1000 Genomes imputation showed higher association signals that exceeded the significance thresholds for pOJIA, but with relatively low imputation quality reflecting the difficulty in imputing rare variants that are only weakly tagged by the genotyped SNPs. These results indicate that the haplotype associations identified in this study are not due to a SNP present in 1000 Genomes imputation analysis but untyped in our present study. Full sequencing of the region in cases and controls will be needed to be performed in order to identify all the variations present in this region.

This study supports our previous finding of significant association of a genetic variant of *IL-20* with sJIA in a cohort of twice the previous cohort size, indicating that this cytokine is biologically important in this disease. It is known that *IL-20* is a potent inflammatory cytokine whose expression can be induced by IL-1β through both p38 MAPK and NF-kB activity [17], and IL-1ß is implicated in pathogenesis of sJIA [18]. The expression of *IL-20* mRNA is predominantly by keratinocytes [19] and activated monocytes [20], [21]. This cytokine has been found to be increased in other inflammatory diseases. High *IL-20* mRNA level is found in the skin of patients with psoriasis [19], [22] and the protein level is high in synovial fluid from patients with rheumatoid arthritis [23]. Other studies have also suggested a potential role of *IL-20* in atherosclerosis and angiogenesis [24], [25]. Overexpression of *IL-20* in mice is lethal secondary to defective skin formation and poor development of lymphoid tissue [26]. Our finding that there is increased risk with the haplotype containing variants of *IL-20* and *MAPKAPK2* suggests that *IL-20* might have interdependent biological activity with *MAPKAPK2* that contribute to disease susceptibility or severity. *MAPKAPK2* is a Ser/Thr protein kinase that is activated through direct phosphorylation by p38 MAP kinase. IL-1β signals through the p38 pathway to induce *IL-20* expression, via *MAPKAPK2* and other signaling molecules, and so genetic variants in these two genes can determine the final outcome of an IL-1ß signal.

Many genetic associations of *IL-10* SNPs have been reported in the literature. A number of SNPs in *IL-10* have been associated with various inflammatory and autoimmune diseases including Behcet’s disease [27], non-infectious uveitis [28], severity of Rheumatoid Arthritis [29], and Type 1 diabetes [30]. The significant tSNPs found in this study for *IL-10* are located within a well characterised co-coordinately regulated locus containing several DNase hypersensitive sites identified in murine T cell populations and bone marrow-derived macrophages [6], [31]. Therefore the findings of haplotype association within the *IL-10* gene locus as well as association of some of the SNPs with lower expression are interesting, and further work is needed to see if these contribute significant risk.

Two *IL-10* genetic variants (rs1878672 and rs1800896) showing some evidence of association with sJIA and pOJIA at single point analysis decreased *IL-10* mRNA expression as suggested by data obtained from the gene expression HapMap database. This biological function suggests that the associations of these variants with disease are less likely to be false positives. However, functional extrapolation from expression databases should be interpreted contextually, taking into account that the expression data is for B cells and the genotype data is from four different populations. Tissue specific expression will need to be explored once we have identified the real disease associated gene variants from sequencing.

Of great interest is that fine mapping of the extended *IL-10* gene family region revealed for the first time association of eOJIA with tSNPs from regions surrounding *FCAMR* and *C1orf116*, an interesting region where chromatin modeling activity has been described by the ENCODE (EnCyclopedia of DNA Elements) project (available on UCSC genome browser). In addition, we have shown some evidence that tSNPs rs10863962 and rs2353550 increases *FCAMR* mRNA expression in transformed B cells from HapMap expression database, suggesting functional roles for the associated genetic variants.

In summary, we have applied three different statistical methods to this data set (single SNP analysis, HyperLasso, haplotype analysis), each having different strengths with the aim of optimising our ability to determine the genetic architecture of JIA in the IL10 region. None of these approaches produced highly significant results or easily interpretable results apart from the single point association with IL-20 in sJIA, and larger sample sizes will be needed to follow up on these findings. Despite these caveats, we have observed differences in the association of *IL-10* gene family with subtypes of JIA. We have provided supporting evidence for previous published associations of genetic variants with sJIA. We have shown that haplotype analyses provide stronger signals than single point analysis which is consistent with synthetic association [15]. The identification of rare, risk haplotypes represents only one possibility for synthetic association and direct sequencing must be performed to explore this further.

This comprehensive candidate gene association study covering all known SNPs with the tSNP approach is the first to be performed for the *IL-10* family, in the largest cohort of a rare disease. It has revealed biologically relevant novel genetic associations that will require further characterisation with gene sequencing to identify the real disease associated genetic variations.

## Materials and Methods

### Ethics Statement

Ethical approval for the study was obtained from Great Ormond Street Hospital for Children NHS Trust and Institute of Child Health, Research Ethics Committee (reference 02RU06 and 04RU07 (05/Q0508/95)) and parents provided written informed consent.

### Patient and Control Samples

Patients with JIA were included from Great Ormond Street Hospital (GOSH), University College London Hospital, Wexham Park Hospital, the British Society for Paediatric and Adolescent Rheumatology (BSPAR) National Repository for JIA and the Childhood Arthritis Prospective Study (CAPS) repository, both housed at the Arthritis Research UK Epidemiology Unit in Manchester, and the SPARKS Childhood Arthritis Response to Medication (CHARMS) study housed at the UCL Institute of Child Health, London. Patients were classified according to the criteria of the International League of Associations for Rheumatology [1]. The sJIA patients in this present study are an expanded population containing 122 patients (55.7%) from Fife et al [9]. The oligoarticular JIA samples are a different cohort from patients previously studied by Crawley et al [7].

Genotype data for the control population was downloaded from the WTCCC web site at http://www.wtccc.org.uk [32]. After matching and removing duplicated individuals, the WTCCC2 cohorts comprised 2889 samples from the 1958 British Birth Cohort (1958BC) and 2834 samples from the UK Blood Services collection (NBS) that were used as our controls. They were genotyped on both the Illumina 1.2 M Duo (Human1-2M-DuoCustom_v1) chip and the Affymetrix v6.0 chip.

### Selection of Tagging SNPs for Typing

We used two data sources in order to obtain thorough SNP coverage of the candidate genes: 1) the International HapMap project [33] for the CEPH (Utah residents with ancestry from northern and western Europe) population (HapMap data release #24/phaseII Nov07, on NCBI B36 assembly, dbSNPb126) and 2) the population of European descent from SeattleSNPs which is part of the NHLBI Programs for Genomic Applications (PGA) (www.nhlbi.nih.gov/resources/pga) that have re-sequenced genes underlying inflammatory diseases in humans. We used the Haploview programme (version 3.2) [34] to calculate linkage disequilibrium between SNPs with minor allele frequency (MAF) of 0.1. We then selected SNPs that tagged other SNPs with the criteria of minimum r^2^ value of 0.8. The selected SNP set with successful assay design consisted of 68 tSNPs that captures 290 SNPs spanning the region 204868570–205258803 bp (390.2 Kb) of chromosome 1. The cut-off for this region was chosen at the end of a block of linkage disequilibrium (LD) that also corresponded to region of conserved sequences between different mammals identified using homology VISTA plots. The rationale for inclusion of conserved regions is that such regions can contain important regulatory elements for the genes. As a result of this approach, 6 flanking genes that are not members of *IL-10* gene cluster were also included: dual-specificity tyrosine-(Y)-phosphorylation regulated kinase 3 (*DYRK3*), mitogen-activated protein kinase-activated protein kinase 2 (*MAPKAPK2*), both located downstream of *IL-10*, and Fas apoptotic inhibitory molecule 3 (*FAIM3*), polymeric immunoglobulin receptor (*PIGR*), receptor for Fc fragment of IgA and IgM (*FCAMR*), and chromosome 1 open reading frame (*C1orf116*) all upstream of *IL-10*.

### Genotyping

Genotyping of the cases in this study was performed with the Illumina GoldenGate assay genotyping platform (custom 384 SNP panel, 96-sample Sentrix array matrix). To check the quality of the genotyping, automatic clustering of the samples and genotype calling was done with the Illumina BeadStudio software (version 3.2.23) and confirmed independently by two investigators. Duplicates of 28 individuals were included for quality control purposes.

### Association Analysis

Single point, allelic association analysis of the genotyping data was performed using the software PLINK [35], conditional and haplotype analyses were performed with the software UNPHASED [36].

Imputation analysis, based on the 1000 Genomes Project data (Interim Phase I Haplotypes; 2010–11 data freeze; 2011-06 haplotypes), was carried out using the phased data generated at the Baylor College of Medicine. This release includes haplotypes for a total of 1,094 individuals, from which we used in our analysis the 381 Caucasian. Data for the chromosome 1 region of interest, plus additional 250 Kb on each side (206551.9–207442.2 Kb, based on Build 37) were extracted, for a total number of 10,765 SNPs present in the region (http://www.sph.umich.edu/csg/abecasis/MACH/download/1000G-PhaseI-Interim.html).

We performed genotype imputation using a hidden Markov model algorithm implemented in MACH software version 1.0 [37], [38]. Parameters for the hidden Markov model for imputation were first estimated in the reference set, and then all individuals were imputed based on those parameters. A dosage score (ranging from 0 to 2) was computed at each SNP for each individual, which is the expected number of copies of a given allele conditional on the genotypes of directly assayed SNPs and integrating overall possible configurations of the phased reference haplotypes. Test for association were carried out using *mach2dat* that performs logistic regression using imputed SNP dosage data. Only those SNPs with a predicted imputation quality (Rsq) value of at least 0.3 were considered, resulting in 1,327 SNPs.

In order to identify a subset of SNPs that best predicts disease status among all imputed and typed SNPs, we run HyperLasso that implements an algorithm for finding approximate modes of a penalised likelihood function for linear or logistic regression [39]. HyperLasso is useful when there are many more predictors than observations, because the penalty function can overcome the problem of over-fitting that would undermine any usefulness of standard logistic regression. The method selects a subset of SNPs that best predicts disease status, while controlling the type-I error of the selected SNPs. We used the Normal-Exponential-Gamma (NEG) probability density penalty function and the values of the shape and scale parameters were determined by a permutation strategy (100 iterations), as in Vignal et al [40] in order to identify the largest posterior mode within each dataset. This method avoids some of the pitfalls of forward- or backward selection methods but despite its statistical strengths it has not yet been widely applied in genetic studies.

Finally, to explore more complex patterns of association with SNPs that are poorly tagged by tSNPs, an iterative conditional analysis procedure was implemented for each JIA subtype. This strategy distinguishes between tSNPs showing association through LD and builds multi-SNP models where SNPs contributing independent association signals. Conditional analysis uses a baseline model of specific SNP, then tests whether including an additional SNP in the model significantly improves fit of the model. The method therefore assesses whether the additional SNP contributes further information for association, after the association at the first SNP (or SNPs) is accounted for. For the first conditional analysis, we conditioned on the tSNP with the strongest evidence of association, testing all other tSNPs for association in the presence of this tSNP. If any tSNPs achieved suggestive significance (p≤0.0189, see below), the most significant of these tSNPs was included in the conditional part of the model, and the analysis repeated to identify any further tSNPs contributing independent association effects. This procedure was repeated until no tSNP achieved suggestive significance threshold. For tSNPs included in the final model, we tested the fit of a SNP model (a joint effect, assuming independent contributions to risk from each tSNP), and a haplotype model, where interactions between SNPs are modelled.

Conditional analysis was performed in UNPHASED [35] under a full haplotype model omitting rare haplotypes with frequency <1%. This model estimates separate risk effects (OR) for each haplotype, and gives a p-value for the improvement in fit of the model with the additional SNP, compared to the baseline model containing only conditional SNP(s). For tSNPs in the final conditional analysis, the SNP-based model was fitted using ‘allele main effect’ model in UNPHASED, which constrains the haplotype effect to be equal to the product of effect for each tSNP allele. To determine the most parsimonious well-fitting model, we tested for a significant difference in fit between the haplotype model (degrees of freedom = number of haplotypes −1) and the SNP model (degrees of freedom = number of SNPs −1). Empirical p-values were also calculated for haplotype-based tests by running 100,000 permutations generated by randomly shuffling the affection status between cases and controls with UNPHASED.

### Significance Levels Accounting for Multiple Testing

Applying an appropriate correction for multiple testing is essential for a candidate gene study to be interpreted correctly. In this study, levels of significance were applied as appropriate for the different analysis strategies used. For the single-point analysis of 53 SNPs, Nyholt’s method [10] was used to calculate the effective number of tests within the region based on the LD pattern in the WTCCC2 controls. The 53 tSNPs were equivalent to 30.9 independent tests, and a Bonferroni corrected p-value is then calculated from 0.05/30.9 = 0.00166. A similar method was applied in the imputation analysis; imputed SNPs gave an equivalent of 320 independent tests and a p-value of 0.00016.

For the exploratory conditional analysis, no equivalent method to determine appropriate correction for multiple testing is available. The most significant SNP from each JIA sub-phenotype was used to seed the conditional analysis. Additional SNPs were added in an iterative procedure if they achieved a threshold of *suggestive* significance, the level expected to arise once in the analysis of 53 SNPs in this region, with p = 0.0189 = 1/53.

### Correlation of Genotype with Gene Expression

We investigated the relationship between mRNA expression and genotype from the transformed B-cell lines dataset generated from a large number of unrelated HapMap individuals [11]. Statistical analysis was performed in SPSS using linear regression to evaluate the association between normalized log2-transformed gene expression values and genotypes of 10 tSNPs with high risk of disease alleles (OR >1) from the single SNP analysis (Table 1). A Bonferroni correction was applied to determine significant correlation between genotype and gene expression correcting for 12 analyses, giving a significance threshold of p<0.0042 (two isoforms were available for *MAPKAPK2*).

## Supporting Information

Figure S1 **LD pattern in the WTCCC2.**
(PNG)

Figure S2 **Results of penalised logistic regression using HyperLasso program for typed and imputed SNPs for sJIA, pOJIA, and eOJIA. HyperLasso-detected SNPs are highlighted (HLasso in “red”).**
(TIF)

Table S1 **Results of the imputation analysis. Only SNPs showing at least** ***suggestive*** **significance in any of the sub-phenotypes are shown. In bold are indicated significant SNPs.**
(DOC)
