# Supplementary figures and images for: Association of the IL-10 Gene Family Locus on Chromosome 1 with Juvenile Idiopathic Arthritis (JIA)
Source: PLoS One. 2012 Oct 18;7(10):e47673. doi: 10.1371/journal.pone.0047673 (PMC3475696; doi:10.1371/journal.pone.0047673)

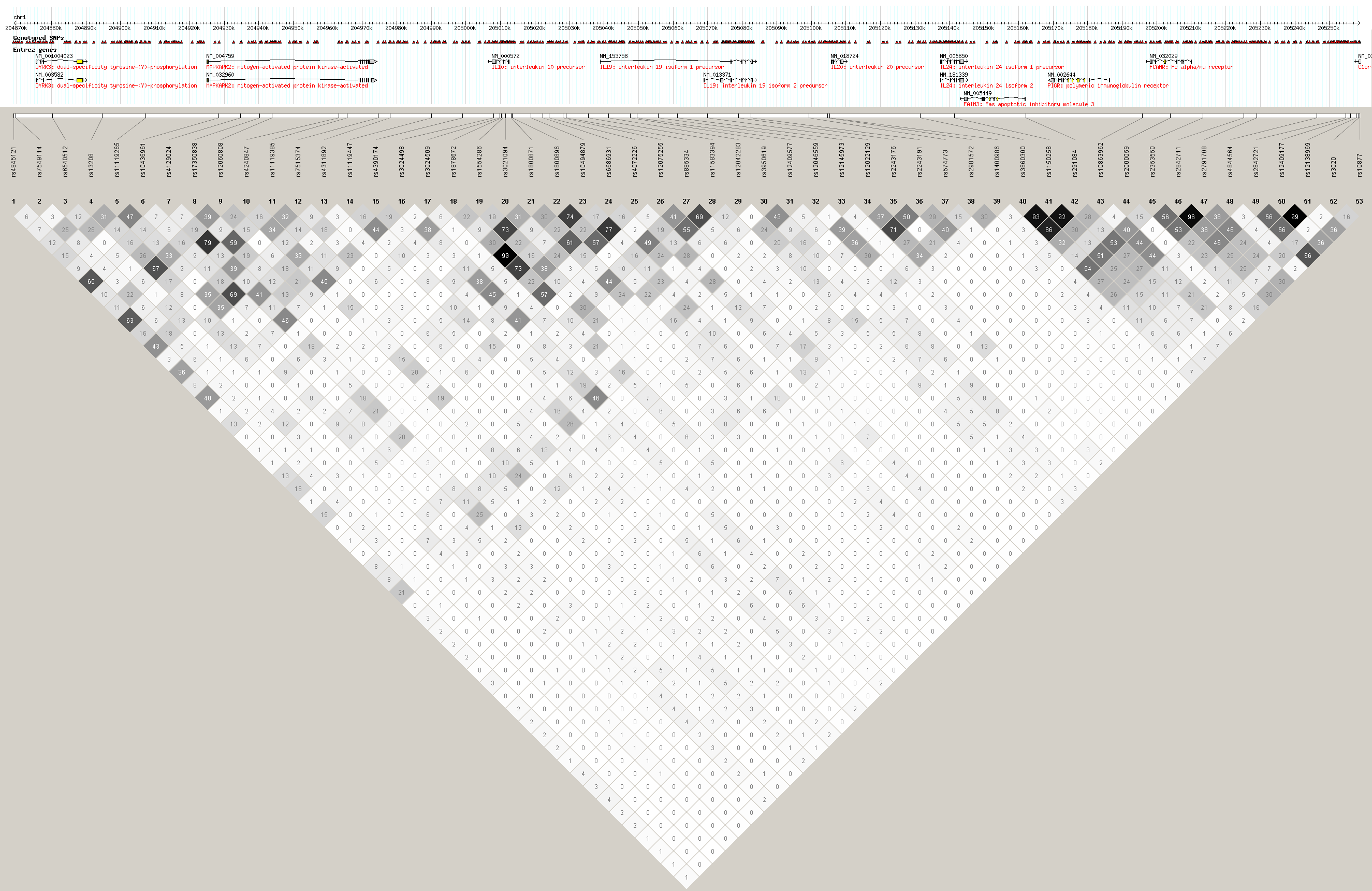

Supplement: Figure S1 — LD pattern in the WTCCC2. (PNG) [file pone.0047673.s001.png]

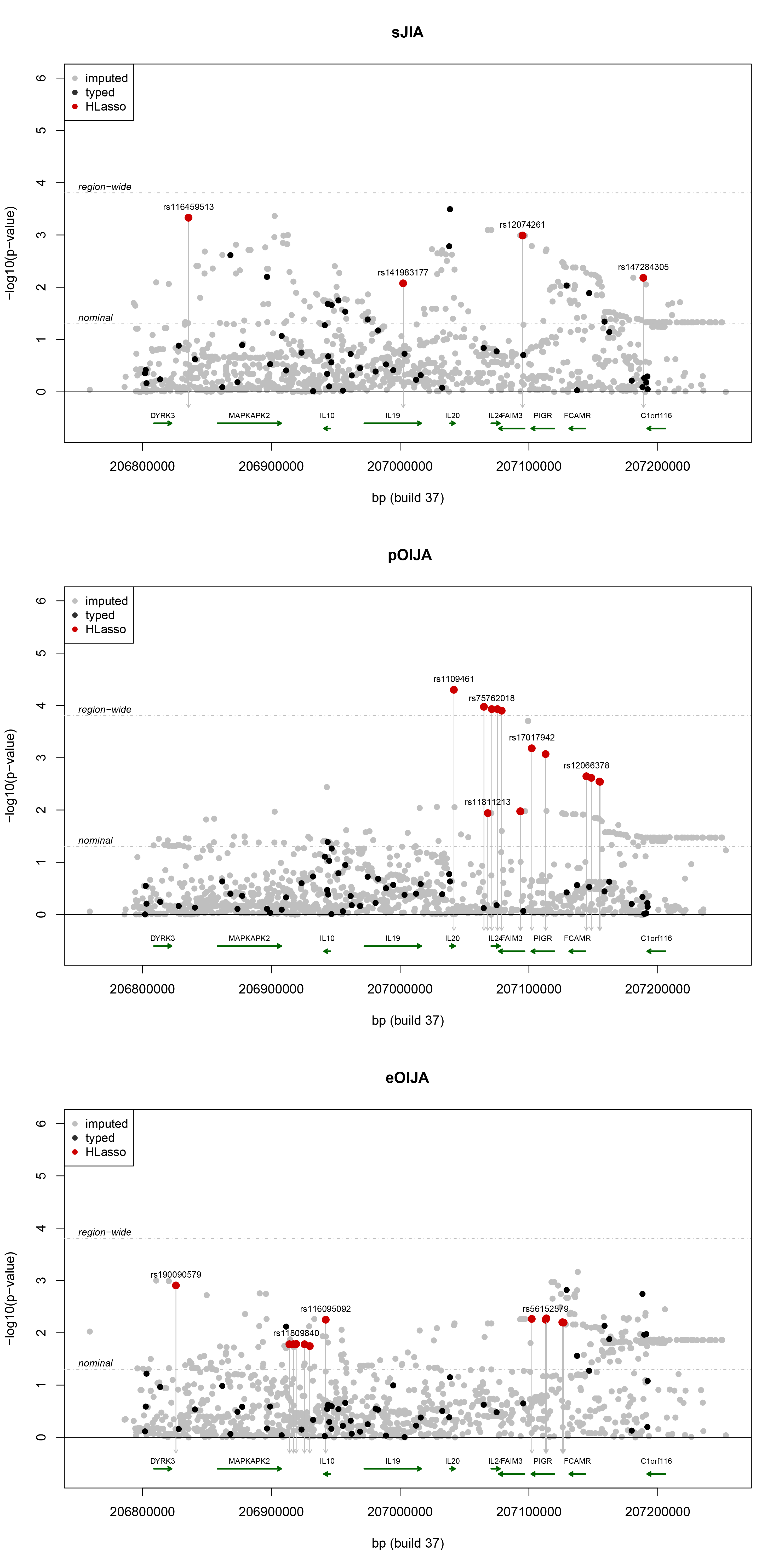

Supplement: Figure S2 — Results of penalised logistic regression using HyperLasso program for typed and imputed SNPs for sJIA, pOJIA, and eOJIA. HyperLasso-detected SNPs are highlighted (HLasso in “red”). (TIF) [file pone.0047673.s002.tif]
