# Supplementary material for: Association of the IL-10 Gene Family Locus on Chromosome 1 with Juvenile Idiopathic Arthritis (JIA)
Source: PLoS One. 2012 Oct 18;7(10):e47673. doi: 10.1371/journal.pone.0047673 (PMC3475696; doi:10.1371/journal.pone.0047673)
Supplement: Table S1 — Results of the imputation analysis. Only SNPs showing at least suggestive significance in any of the sub-phenotypes are shown. In bold are indicated significant SNPs. (DOC) [file pone.0047673.s003.doc]

**Table S1:** Results of the imputation analysis. Only SNPs showing at least *suggestive* significance in any of the sub-phenotypes are shown. In bold are indicated significant SNPs.

| **SNP**  **(1000G)** | **rs#**  **(dbSNP)** | **bp**  **(build 37)** | **alleles** | **MAF (dbSNP)** | **MAF**  **(imp)** | **location** | **GENE** | **Rsq** | **sJIA** | **pOJIA** | **eOJIA** |
| --- | --- | --- | --- | --- | --- | --- | --- | --- | --- | --- | --- |
| chr1:206835771 | rs116459513 | 206835771 | T,C | 0.004 | 0.0002 | -- | -- | 0.57 | 0.000467 | 0.051780 | 0.998900 |
| chr1:206902628 | rs146359022 | 206902628 | A,G | NA | 0.0002 | intron | MAPKAPK2 | 0.69 | 0.000435 | 0.010720 | 0.910700 |
| rs1400986 | rs1400986 | 207038686 | T,C | 0.233 | 0.1558 | Promoter | IL20 | typed | 0.000322 | 0.231600 | 0.070850 |
| rs1109461 | rs1109461 | 207041803 | T,C | 0.076 | 0.0008 | Intron | IL20 | 0.39 | 0.015940 | **0.000050** | 0.007196 |
| rs73089070 | rs73089070 | 207065088 | T,C | 0.025 | 0.0001 | Promoter | IL24 | 0.40 | 0.266300 | **0.000106** | 0.389700 |
| rs11811213 | rs11811213 | 207068016 | T,C | 0.012 | 0.0008 | Promoter | IL24 | 0.52 | 0.000805 | 0.011440 | 0.006612 |
| rs3093417 | rs3093417 | 207070941 | T,C | 0.026 | 0.0008 | UTR | IL24 | 0.52 | 0.000799 | 0.011500 | 0.006612 |
| chr1:207071206 | rs75762018 | 207071206 | C,T | 0.019 | 0.0001 | **L10P** | IL24 | 0.40 | 0.269200 | **0.000118** | 0.392900 |
| rs3093426 | rs3093426 | 207073474 | A,G | 0.07 | 0.0001 | intron | IL24 | 0.41 | 0.267900 | **0.000117** | 0.391500 |
| rs3093432 | rs3093432 | 207075637 | G,A | 0.07 | 0.0001 | intron | IL24 | 0.41 | 0.270300 | **0.000118** | 0.394000 |
| chr1:207078795 | rs143572917 | 207078795 | A,G | NA | 0.0001 | intron | FAIM3 | 0.40 | 0.290900 | **0.000126** | 0.412900 |
| rs1356495 | rs1356495 | 207093202 | T,C | 0.087 | 0.0009 | intron | FAIM3 | 0.55 | 0.001010 | 0.010610 | 0.005475 |
| rs6694769 | rs6694769 | 207093486 | C,T | 0.084 | 0.0009 | intron | FAIM3 | 0.55 | 0.001012 | 0.010630 | 0.005484 |
| rs12074261 | rs12074261 | 207095048 | C,G | 0.084 | 0.0009 | intron | FAIM3 | 0.55 | 0.001019 | 0.010640 | 0.005467 |
| chr1:207097015 | rs115231212 | 207097015 | C,T | 0.052 | 0.0009 | -- | -- | 0.56 | 0.001028 | 0.010540 | 0.005430 |
| chr1:207099171 | rs76132612 | 207099171 | A,C | 0.004 | 0.0001 | -- | -- | 0.38 | 0.564300 | 0.000198 | 0.547500 |
| chr1:207099538 | rs143365537 | 207099538 | C,T | NA | 0.0001 | -- | -- | 0.38 | 0.564300 | 0.000198 | 0.547500 |
| rs17017942 | rs17017942 | 207102290 | C,T | 0.024 | 0.0011 | 3' UTR | PIGR | 0.61 | 0.001616 | 0.000659 | 0.005430 |
| rs56152579 | rs56152579 | 207112999 | C,T | 0.024 | 0.0011 | intron | PIGR | 0.63 | 0.002039 | 0.000848 | 0.005599 |
| rs729040 | rs729040 | 207113592 | C,G | 0.072 | 0.0010 | intron | PIGR | 0.63 | 0.001885 | 0.010420 | 0.005294 |
| rs2153438 | rs2153438 | 207137985 | A,C | 0.334 | 0.2754 | intron | FCAMR | 0.98 | 0.021480 | 0.347600 | 0.000684 |
| rs12066378 | rs12066378 | 207144619 | C,T | 0.096 | 0.0013 | Promoter | FCAMR | 0.70 | 0.005259 | 0.002263 | 0.007676 |
| rs12097910 | rs12097910 | 207148459 | T,G | 0.07 | 0.0013 | Promoter | FCAMR | 0.70 | 0.005723 | 0.002423 | 0.007948 |
| rs73072484 | rs73072484 | 207154828 | G,A | 0.062 | 0.0014 | -- | -- | 0.69 | 0.007620 | 0.002855 | 0.008542 |
| rs12073181 | rs12073181 | 207155247 | G,A | 0.063 | 0.0014 | -- | -- | 0.69 | 0.007649 | 0.002866 | 0.008549 |
